# Gdnf Acts as a Germ Cell-Derived Growth Factor and Regulates the Zebrafish Germ Stem Cell Niche in Autocrine- and Paracrine-Dependent Manners

**DOI:** 10.3390/cells11081295

**Published:** 2022-04-11

**Authors:** Lucas B. Doretto, Arno J. Butzge, Rafael T. Nakajima, Emanuel R. M. Martinez, Beatriz Marques de Souza, Maira da Silva Rodrigues, Ivana F. Rosa, Juliana M. B. Ricci, Aldo Tovo-Neto, Daniel F. Costa, Guilherme Malafaia, Changwei Shao, Rafael H. Nóbrega

**Affiliations:** 1 Reproductive and Molecular Biology Group, Department of Structural and Functional Biology, Institute of Biosciences, São Paulo State University (UNESP), São Paulo 18618-689, Brazil; lucas.doretto@unesp.br (L.B.D.); julianobutzge@gmail.com (A.J.B.); nakajimatakahiro.r@gmail.com (R.T.N.); erm_martinez@yahoo.com.br (E.R.M.M.); bia.mrqssza@gmail.com (B.M.d.S.); maira.rodrigues@unesp.br (M.d.S.R.); ivana.felipe@unesp.br (I.F.R.); ju1716@hotmail.com (J.M.B.R.); aldotovo@gmail.com (A.T.-N.); fernandesdacostadaniel@gmail.com (D.F.C.); guilhermeifgoiano@gmail.com (G.M.); 2 Biological Research Laboratory, Goiano Federal Institution—Urata Campus, Rodovia Geraldo Silva Nascimento, 2.5 km, Zona Rural, Urutaí 75790-000, Brazil; 3 Key Lab of Sustainable Development of Marine Fisheries, Ministry of Agriculture and Rural Affairs, Yellow Sea Fisheries Research Institute, Chinese Academy of Fishery Sciences, Qingdao 266072, China; shaochangwei303@163.com; 4 Laboratory for Marine Fisheries Science and Food Production Processes, Qingdao National Laboratory for Marine Science and Technology, Qingdao 266071, China

**Keywords:** Gdnf, Gfrα1, spermatogonial stem cell, spermatogenesis, zebrafish

## Abstract

Glial cell line-derived neurotrophic factor (GDNF) and its receptor (GDNF Family Receptor α1-GFRα1) are well known to mediate spermatogonial stem cell (SSC) proliferation and survival in mammalian testes. In nonmammalian species, Gdnf and Gfrα1 orthologs have been found but their functions remain poorly investigated in the testes. Considering this background, this study aimed to understand the roles of the Gdnf-Gfrα1 signaling pathway in zebrafish testes by combining in vivo, in silico and ex vivo approaches. Our analysis showed that zebrafish exhibit two paralogs for Gndf (*gdnfa* and *gdnfb*) and its receptor, Gfrα1 (*gfrα1a* and *gfrα1b*), in accordance with a teleost-specific third round of whole genome duplication. Expression analysis further revealed that both ligands and receptors were expressed in zebrafish adult testes. Subsequently, we demonstrated that *gdnfa* is expressed in the germ cells, while Gfrα1a/Gfrα1b was detected in early spermatogonia (mainly in types A_und_ and A_diff_) and Sertoli cells. Functional ex vivo analysis showed that Gdnf promoted the creation of new available niches by stimulating the proliferation of both type A_und_ spermatogonia and their surrounding Sertoli cells but without changing *pou5f3* mRNA levels. Strikingly, Gdnf also inhibited late spermatogonial differentiation, as shown by the decrease in type B spermatogonia and down-regulation of *dazl* in a co-treatment with Fsh. Altogether, our data revealed that a germ cell-derived factor is involved in maintaining germ cell stemness through the creation of new available niches, supporting the development of spermatogonial cysts and inhibiting late spermatogonial differentiation in autocrine- and paracrine-dependent manners.

## 1. Introduction

GDNF (Glial cell line-derived neurotrophic factor) is a closely related member of the TGF-β superfamily which belongs to the GDNF family of ligands (GFLs). This family of ligands consists of Gdnf, neurturin, artemin and persephin [1]. The importance of GDNF for spermatogonial stem cell (SSC) maintenance was unveiled by Meng et al. [2], who demonstrated that mice with impaired GDNF signaling exhibited a progressive loss of SSCs, whereas GDNF overexpression promoted germ cell hyperplasia and ultimately tumors [2]. Further studies showed that GDNF promoted in vitro expansion of mouse germline stem cells [3,4], this being considered an indispensable factor for long-term culture of SSCs for several species of rodents [3,5,6]. More recently, experiments using mice that ectopically expressed stage-specific GDNF in Sertoli cells revealed that GDNF increased SSC self-renewal by blocking differentiation rather than actively stimulating their proliferation [4]. Altogether, these studies in mammals demonstrated that GDNF is an important factor for SSC self-renewal, proliferation of the stem cell direct progenitors and maintenance of the SSC undifferentiated state (see the review in Parekh et al. [7]; see also Mäkelä and Hobbs [8]).

GDNF signaling occurs through binding the non-signaling co-receptor of the GDNF Family Receptor α1 (GFRα1), which is attached to the cell membrane by glycosylphosphatidylinositol-anchors [1]. The complex GDNF-GFRα1 associates with a single transmembrane RET receptor tyrosine kinase, leading to the activation of RET’s intracellular kinase domain and the subsequent stimulation of different downstream cellular pathways [1]. In mammalian testes, GDNF is produced by testicular somatic cells, including Sertoli cells [2,9,10], peritubular myoid cells under the influence of androgens [11,12] and testicular endothelial cells, which seem to be the major GDNF-producing sources in mouse testes [13]. In rodents, GFRα1 is present in a subpopulation of single type A spermatogonia (A_s_), which also expresses the inhibitor of DNA binding 4 (ID4) [14,15]. This subpopulation is considered the purest functional SSC population [14,15]. However, several other studies have demonstrated that GFRα1 is not exclusively detected in SSCs but is also expressed in types A paired (A_pr_) and aligned (A_al_) spermatogonia [16,17,18,19,20]. Similar expression patterns have been reported in other mammalian species, such as hamsters [21], pigs [22], collared peccaries [23,24], buffaloes [25], different equine species [26] and primates, including humans [27,28].

In nonmammalian species, particularly in fish, Gdnf/Gfrα1 homologs have been found in a limited number of species, such as dogfish (*Scyliorhinus canicula*) [29], rainbow trout (*Oncorhynchus mykiss*) [30,31,32] and medaka (*Oryzias latipes*) [33]. In these species, Gdnf and Gfrα1 are co-expressed in type A undifferentiated spermatogonia, suggesting an autocrine mechanism for Gdnf-mediated functions in fish testes [30]. The physiological relevance of Gdnf for type A undifferentiated spermatogonia has been further demonstrated by in vitro studies showing that recombinant human GDNF (rh GDNF) promoted the proliferation and long-term maintenance of dogfish spermatogonia with stem characteristics [29]. Similar findings were reported by Wei et al. [34], who showed that two Gdnf homologs in medaka, named Gdnfa and Gdnfb, stimulated proliferation of SG3, a medaka spermatogonial stem cell line. On the other hand, studies in rainbow trout revealed that *gdnfb* mRNA levels increased during the arrest of the spermatogenic cycle (end of germ cell proliferation and differentiation), suggesting that Gdnfb is likely involved in the repression of SSC differentiation rather than proliferation [31]. Considering this background and the lack of knowledge about Gdnf-Gfrα1 signaling in fish, this study aimed to unravel the autocrine/paracrine roles of Gdnf on the zebrafish germ stem cell niche and to expand our knowledge about the critical factors involved in SSC activity as well as improve our abilities to predict the consequences of changes involved in the physiological mechanisms related to Gdnf. To these ends, we initially performed phylogenetic and synteny analyses for Gfrα1 and then investigated the testicular expression profiling of *gdnf* (*gdnfa* and *gdnfb*) and *gfrα1* (*gfrα1a* and *gfrα1b*) transcripts in zebrafish testes. Subsequently, we identified the cellular types expressing Gdnf and Gfrα1 and assessed the biological effects of Gdnf through an ex vivo testis culture system. According to Oatley and Brinster [35], the impairment of SSC function disrupts spermatogenesis and causes subfertility or infertility in males; therefore, knowing the mechanisms that regulate SSC homeostasis is imperative for the conservation of species or for their use as experimental models in studies focusing on the treatment of pathological conditions affecting the reproductive organs in humans.

## 2. Material and Methods

### 2.1. Zebrafish Stocks

Sexually mature zebrafish (*Danio rerio*, outbred) (4–5 months old) were kept in 6 L water tanks in a recirculating system with controlled photothermal conditions (27 °C and 14 h of light and 10 h dark). Parameters such as salinity, pH, dissolved oxygen and ammonia were monitored daily in all tanks. Fish were fed twice a day using commercial food (Zeigler^®^, Gardners, PA, USA). Handling and experimentation were in accordance with Brazilian legislation regulated by the National Council for the Control of Animal Experimental (CONCEA) and the Ethical Principles in Animal Research of São Paulo State University (protocol no. 666-CEUA). Zebrafish is a tropical freshwater fish species natural to rivers in Southern Asia [36,37,38] and has been considered a versatile model for reproductive biology [39], besides being used as a model for translational research in human health and disease [40]. Therefore, these aspects justify the choice of this species in our study.

### 2.2. Sequence Analysis

The predicted amino acid sequences for Gfrα1a and Gfrα1b of *D. rerio* (Q98TT9 and Q98TT8, respectively), GFRA1 of *Homo sapiens* (P56159), *Rattus norvegicus* (Q62997) and *Mus musculus* (P97785) were obtained from the Universal Protein Resource (UniProt, accessed 09/12/2019) and aligned using the MEGA algorithm allocated in Geneious Pro 4.8.5 software [41]. For the phylogenetic analysis, we retrieved the protein sequences for GFRα1 (Gfrα1a and Gfrα1b) from the Universal Protein Resource (UniProt, accessed on 25 February 2020), the National Center for Biotechnology Information (NCBI, 25 February 2020) and Ensembl (accessed on 25 February 2020 [42]). For this analysis, we retrieved vertebrate sequences for GFRα1 and Growth arrest-specific protein 1 (GAS1) from humans (GAS1 as an outgroup). The predicted amino acid sequences were aligned using the Muscle algorithm [43] allocated in Geneious Pro 4.8.5 software [41]. The choice of the best fitting model of evolution was performed with SMS [44]. Phylogenetic reconstruction was determined by Bayesian methods implemented in Beast v1.7.0 software [45]. This step was carried out according to Geraldo et al. [46], with adaptations. Branch values were supported by posterior probabilities obtained by Bayesian analysis. For Bayesian methods, the burn-in was obtained through Tracer [45] using log likelihood scores, and data were compiled in TreeAnnotator [45] after trees that were out of the convergence area had been discarded. The visualization and the final tree edition were generated using FigTree v1.3.1 [45]. In the phylogenetic analyses, the proportion of invariable sites and γ-distributed rate variation across sites were estimated, and the substitution of rate categories set in four categories. The parameter settings used to reconstruct the phylogeny are shown in Table S2. To construct the synteny regions of *GFRA1* (human), *Gfrα1* (rat and mouse), *gfrα1a* and *gfrα1b* (zebrafish), we used the GenBank database, available at the National Center for Biotechnology Information (NCBI; http://www.ncbi.nlm.nih.gov/) (accessed on 25 February 2020) and Ensembl [42].

### 2.3. Expression Profiling of Gdnf (gdnfa and gdnfb) and Gfrα1 (gfrα1a and gfrα1b) Transcripts in Zebrafish Testes

To investigate the expression of *gdnfa* (glial cell-derived neurotrophic factor a), *gdnfb* (glial cell-derived neurotrophic factor b), *gfrα1a* (gdnf family receptor alpha 1a) and *gfrα1b* (gdnf family receptor alpha 1b) in zebrafish testes, total RNA from testes (*n* = 4 males) was extracted using an RNAqueous^®^-Micro kit (Ambion, Austin, TX, USA), following the manufacturer’s instructions. cDNA synthesis and quantitative reverse transcription polymerase chain reaction (RT-qPCR) were performed as previously described [47]. The number of amplification cycles (Ct-cycle threshold) for *gdnfa*, *gdnfb*, *gfrα1a* and *gfrα1b* were determined through a StepOnePlus™ Real-Time PCR System (Thermo fisher, Waltham, MA, USA, EUA). Primers (Table 1) were designed based on zebrafish sequences available from the Genbank database.

### 2.4. Differential Plating Method

To obtain testicular cellular fractions (germ or somatic cell-enriched fractions), a differential plating method was carried out as previously described by Hinfray et al. [51]. To this end, testes (*n* = 20 males) were digested with 0.2% collagenase (Sigma Aldrich, San Luis, MI, USA) and 0.12% dispase (Sigma Aldrich, San Luis, MI, USA) [47]. Total cell suspension was submitted to a differential plating method, in which somatic cells adhere to the bottom of the plate, whereas germ cells either remain in suspension or only weakly associate with adhering somatic cells [51]. By using this approach, germ and somatic cell-enriched fractions can be obtained [51]. RNA from cell suspensions (total, germ and somatic cell-enriched fractions) was obtained using a PureLink^®^ RNA Mini Kit (Ambion, Austin, TX, USA), following the manufacturer’s instructions. cDNA synthesis was conducted using a SuperScript^®^ II Reverse Transcriptase kit (Invitrogen, Carlsbad, CA, USA) and random hexamers. The relative mRNA levels of *pou5f3* (POU domain, class 5, transcription factor 3) (spermatogonia marker), *vasa* (spermatogonia marker), *gdnfa*, *igf3* (insulin-like growth factor 3) (Sertoli cell marker*),* *gfrα1a* and *gfrα1b* were determined by qRT-PCR. *β-actin* and *ef1* were used as housekeeping genes. The quantification cycle (Cq) values were determined in a StepOne system (Life Technologies, Carlsbad, CA, USA) using SYBR Green (Invitrogen, Carlsbad, CA, USA) and specific primers (Table 1), as described in Section 2.3.

### 2.5. Immunofluorescence and Western Blot

**Figure 1:**
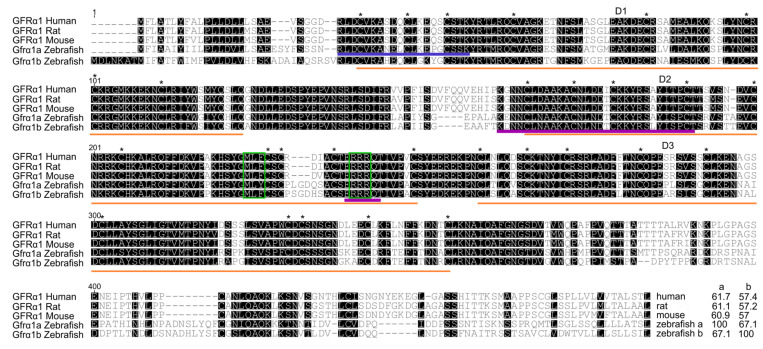
GFRα1 predicted amino acid sequence alignment. Numbers at the top left of the sequences indicate amino acid positions, dashes indicate deletions and black boxes indicate shared sequences. The three cysteine-rich domains (D1–D3) (orange lines), 28 cysteine residues (*) (plus 2 in the terminal region) and two triplets (MLF and RRR) (green boxes) are highly conserved among humans, rodents and zebrafish. At the end of the alignment are the percentage identity values of zebrafish Gfrα1a and Gfrα1b in relation to the other corresponding sequences. The blue line indicates the amino acid sequence recognized by the zebrafish Gfrα1a antibody used in this study; the purple line indicates the putative motifs critical for binding to GDNF.

Testes (*n* = 10 males) were fixed with 4% paraformaldehyde in PBS (Phosphate Buffered Saline) (1X, pH 7.4) for 1 h, embedded in paraplast (Sigma Aldrich, San Luis, MI, USA) and sectioned at 5 μm thickness. After deparaffinization and rehydration, sections were submitted to antigen retrieval by heating slides in sodium citrate buffer (10 mM sodium citrate, 0.05% Tween 20, pH 6.0) until temperatures reached 95–100 °C in a microwave. To reduce background fluorescence, slides were incubated with NaBH4 (sodium borohydride—0.01g dissolved in 1 mL of distilled water) (Sigma Aldrich, San Luis, MI, USA) for 3 min. Subsequently, slides were rinsed with 1X PBS (pH 7.4) and incubated with the biotinylated primary antibody rabbit anti-zebrafish Gfrα1a (1:300, 1X PBS pH 7.4) at 4 °C overnight. Zebrafish polyclonal biotinylated antibody anti-Gfrα1a was synthesized by Rheabiotech (Campinas, SP, Brazil) using the specific antigen sequence ‘RLDCVKANELCLKEPGCSSK’ located at the N-terminus of zebrafish Gfrα1a (Figure 1). This antibody is also potentially able to recognize other Gfrα1 isoforms, such as GFRA1 in humans and rodents and Gfrα1b in zebrafish (Figure 1). After rising, the slides were incubated with Dylight 488 Streptavidin (BioLegend^®^, San Diego, CA, USA) (1:400) or Alexa Fluor 594 Streptavidin (BioLegend^®^, San Diego, CA, USA) (1:400) in 1X PBS (pH 7.4) for 60 min at room temperature. Subsequently, sections were counterstained with Hoechst (1:2000, 1X PBS pH 7.4) (Invitrogen, Carlsbad, CA, USA) or Propidium iodide (PI) (BioLegend^®^, San Diego, CA, USA) (1 mg/mL dissolved in distilled water) and mounted with ProLong Gold Antifade (Thermo Fisher Scientific, Waltham, MA, USA). Control sections were prepared by preadsorbing the zebrafish Gfrα1a antibody with the corresponding peptide (10 μg/1 µL of antibody, Rheabiotech, Campinas, SP, Brazil) or by omitting the primary antibody. Slides were photographed using a Leica SP5 laser scanning confocal microscope (Leica, Wetzlar, Hessen, Germany) from the Electron Microscopy Center, Institute of Biosciences, São Paulo State University (Botucatu, Brazil), and germ cells were classified according to Leal et al. [52].

For the Western blot analysis, testes (*n* = 10 males) were homogenized in an extraction TBST buffer (10 mM Tris–HCl, pH 7.5; 150 mM NaCl; 0.1% Tween 20) containing a cocktail of protease inhibitors (Roche Applied Science, Mannheim, Germany). Subsequently, the homogenate was incubated on ice for 15–20 min before sonication (3 × 1 min on ice) and centrifuged at 4000 rpm at 4 °C for 20 min in order to determine the total protein concentration by means of a NanoVue spectrophotometer (GE Healthcare, Chicago, IL, USA). A total of 40 µg protein was analyzed by sodium dodecyl sulfate polyacrylamide gel electrophoresis (SDS-PAGE). Protein extracts were blotted onto a nitrocellulose membrane (Amersham, Little Chalfont, UK) blocked with 3% non-fat milk diluted in 1X Tris-buffered saline (TBS) (150 mM NaCl, 50 mM Tris-HCl, pH 7.6.) for 1 h, and incubated with the primary antibody rabbit anti-zebrafish Gfrα1a (1:500, Rheabiotech, Campinas, SP, Brazil) at 4 °C overnight. The membrane was washed with TBS and incubated with horseradish peroxidase-conjugated anti-rabbit IgG (1:5000, Santa Cruz Biotechnologies, Dallas, TX, EUA) for 2 h. After washing, blots were developed with a chemiluminescence substrate kit (Pierce ECL Western Blotting Substrate, GE Healthcare, Chicago, IL, USA) and the signal was captured by a CCD camera (ImageQuant LAS 4000 mini^®^, GE Healthcare, Chicago, IL, USA). As controls, some membranes were alternatively incubated with primary antibodies that had been preadsorbed with the respective peptides.

### 2.6. Recombinant Human GDNF

To evaluate the effects of Gdnf on zebrafish spermatogenesis (see below), a rhGDNF purchased from PeproTech^®^ (London, UK) (reference no. 450-10; https://www.peprotech.com/en/recombinant-human-gdnf#productreviews)(accessed on 20 February 2020) was used. We used a recombinant human hormone because the recombinant zebrafish Gdnf is not commercially available. In addition, rhGDNF has previously been used in fish [53]. The rhGDNF was dissolved in sterile Lebovitz medium (L-15) (Sigma-Aldrich, St. Louis, MO, USA) at a concentration of 100 µg/mL and subsequently aliquoted and stored at −20 °C until use. After identifying the binding sites between rhGDNF and human GFRA1A, a 3D structure model was built to predict the interaction sites between rhGDNF and zebrafish Gfrα1a (Q98TT9). The 3D protein structure used was obtained through SWISS-MODEL (swissmodel.expasy.org), with multiple target sequences representing different subunits of a hetero-oligomer (hetero-2-2-mer), and the quality of the modeling was analyzed by means of a Ramachandran plot generated with Rampage software [50]. The template (4ux8.1) and the final model were viewed in the software Pymol (the PyMOL Molecular Graphics System, Version 1.8 Schrödinger, LLC).

### 2.7. Testis Tissue Culture

The effects of rhGDNF on zebrafish spermatogenesis were investigated using a previously established ex vivo culture system [52]. In this system, one testis (left) was incubated in the presence of rhGDNF (100 ng/mL, based on Gautier et al. [53]) and its contra-lateral (right) in the basal culture medium (L-15). The culture medium was changed every 3 days, and after 7 days, testes were collected for histomorphometrical analysis via a BrdU (bromodeoxyuridine) (Sigma Aldrich, San Luis, MI, USA) incorporation assay and gene expression (RT-qPCR) (see below). Additional cultures were carried out to assess the interaction of Gdnf with Fsh-mediated effects on the zebrafish spermatogonial phase [54]. To this end, zebrafish testes (*n* = 10 males) were incubated with recombinant zebrafish Fsh (rzfFsh) (100 ng/mL [55]) (U-Protein Express B.V; Utrecht, the Netherlands) in the presence or absence of rhGDNF (100 ng/mL) for 7 days. After the culture period, testes were collected for RT-qPCR analysis. For histomorphometry, zebrafish testicular explants (*n* = 10) were fixed in 4% buffered glutaraldehyde at 4 °C overnight, dehydrated, embedded in historesin Technovit 7100 (Wehrheim, Germany), sectioned at 4µm thickness and stained with 0.1% toluidine blue to estimate the frequency of the different germ cell cysts using a high-resolution light microscope (Leica DM6000 BD, Leica Microsystems, Wetzlar, Germany). In this analysis, five histological fields for each animal were randomly selected for counting the frequency of germ cell cysts (type A undifferentiated spermatogonia (A_und_), type A differentiated spermatogonia (A_diff_), type B spermatogonia (SPG B), spermatocytes (SPCs) and spermatids (SPTs)), as previously described [47,52].

To investigate the effects of rhGDNF on germ cell proliferation, BrdU (100 µg/mL) was added during the final 6 h of incubation. After incubation, zebrafish testes (*n* = 10) were fixed at 4 °C overnight in freshly prepared methacarn (60% (v/v) absolute ethanol, 30% chloroform and 10% acetic acid) for 4 h. Subsequently, testes were dehydrated, embedded in Technovit 7100 (Wehrheim, Germany), sectioned at 4 µm thickness and used for BrdU immunodetection, as previously described [47,55]. The mitotic index or BrdU incorporation ratio of types A_und_, A_diff_ and Sertoli cells was determined by counting the BrdU-positive and BrdU-negative cells in a total of 100 cells for the same cellular type, as described previously [47,48,55].

For RT-qPCR, total RNA from testicular explants (*n* = 20 males) was extracted using the same method described in Section 2.3. The relative mRNA levels of *gdnfa*, *gfrα1a*, *gfrα1b*, *amh* (anti-Müllerian hormone), *igf3*, *fshr* (follicle stimulating hormone receptor), *pou5f3*, *dazl* (deleted in azoospermia-like) and *sycp3l* (synaptonemal complex protein 3) were evaluated. The mRNA levels of the targets (Cts) were normalized by β-actin levels, expressed as relative values of basal expression levels, according to the 2^−(ΔΔCT)^ method. Primer sequences are indicated in Table 1.

### 2.8. In Silico Analysis of Putative Regulatory Sequences Upstream Human GDNF, Mouse Gdnf and Zebrafish Gdnfa

To retrieve the putative regulatory sequences of upstream human *GDNF* (NM_000514.4), mouse *Gdnf* (NM_010275.3) and zebrafish *gdnfa* (NM_131732.2), the transcription start site (TSS) was found in the Eukaryotic Promoter Database (EPD), and the promoter regulatory regions (3’ to 5’) were prospected by means of the flanking regions (2000 bp) extracted from NCBI. The cAMP response elements (CRE, four different sequences), the androgen receptor binding site (AR, full and half sequences), several NF-kB-binding sites, N-Box, E-Box, TATA-Box and GC-Box (Table S3) were prospected using sequences described in the literature [7,56,57,58,59,60,61].

### 2.9. Statistical Analyses

Data were initially checked for deviations from variance normality and homogeneity through the Shapiro–Wilk and Bartlett’s tests, respectively. Significant differences between two groups were identified using a paired Student’s *t*-test, at 5% probability. Comparisons of more than two groups were performed with one-way ANOVA followed by Student–Newman–Keuls test, at 5% probability. Graphpad Prism 7.0 (Graphpad Software, Inc., San Diego, CA, USA) was used for the statistical analysis.

## 3. Results

### 3.1. Sequence Analyses, Phylogenetic Tree and Genomic Organization of Zebrafish Gfrα1a and Gfrα1b 

Sequence analysis revealed that both predicted zebrafish Gfrα1a and Gfrα1b have sequence characteristics of Gfrα family members, such as the three cysteine-rich domains (D1-3), 28 cysteine residues (plus 2 in the terminal region), and two triplets (MLF and RRR) in the domain D2 (Figure 1). Sequence alignment of zebrafish Gfrα1a and 1b with different GFRA1s (human and rodent) revealed that the three cysteine-rich domains (D1, D2, D3) are highly conserved among the species, highlighting, in particular, the conserved residues and motifs in the domain D2 critical for binding to GDNF and eliciting downstream cellular pathways (Figure 1). Sequence analyses also demonstrated that zebrafish Gfrα1a and 1b have 67.1% identity to each other, and zebrafish Gfrα1a showed a higher identity with mammalian GFRA1 (61.7%, 61.1% and 60.9% similarity to human, rat and mouse GFRA1, respectively) than zebrafish Gfrα1b (57.4%, 57.2% and 57% identity to human, rat and mouse GFRA1, respectively) (Figure 1).

**Figure 2:**
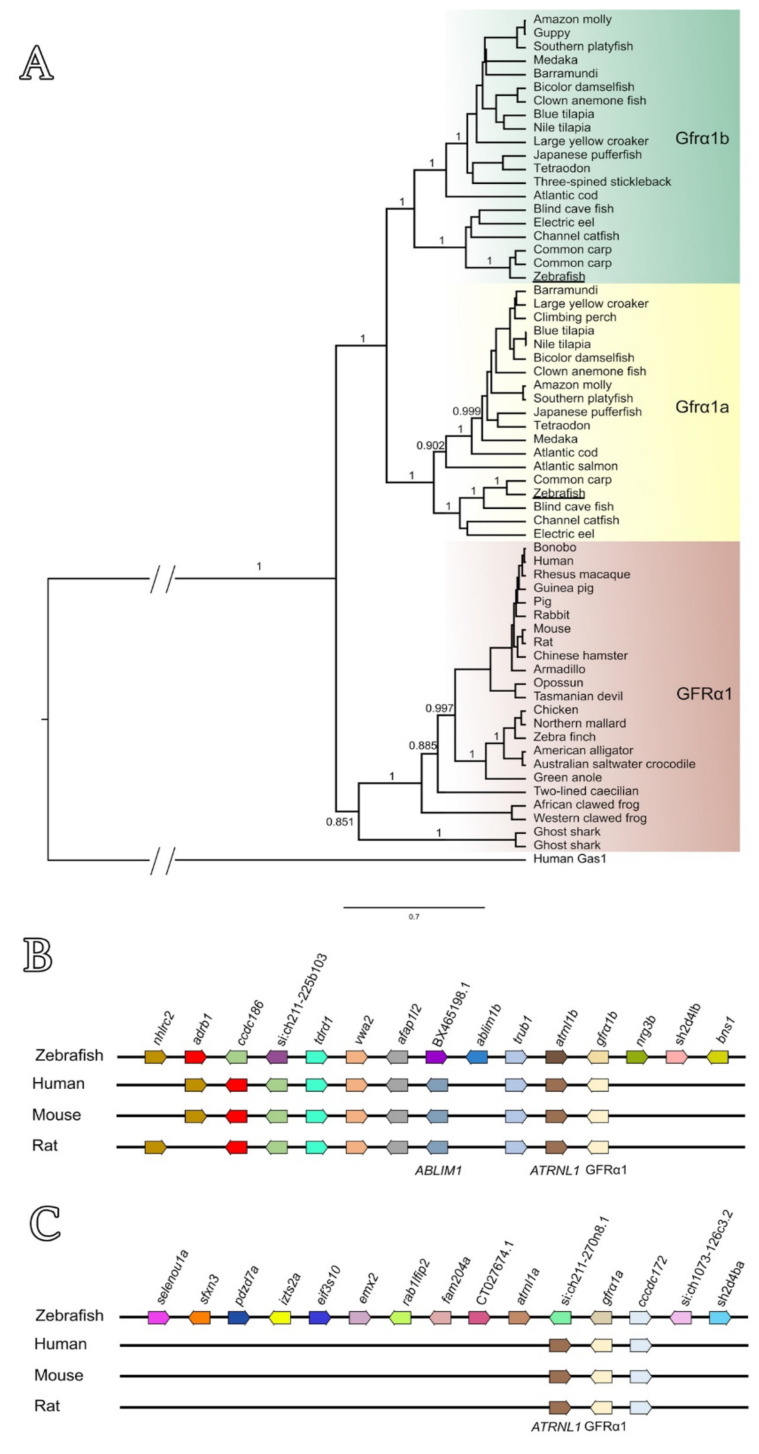
(**A**) Phylogenetic analysis of GFRα1 predicted amino acid sequences across vertebrates. Zebrafish Gfrα1a and 1b (both underlined) are clustered with other fish-specific Gfrα1a (yellow box) and Gfrα1b (green box) sequences, respectively, forming two separate subclades. Note that the GFRA1 sequences from other vertebrates (mammals, birds, reptiles, amphibians and Chondrichthyes) formed a separate clade (brown box). Branch values represent posterior probabilities obtained by Bayesian analysis (see Table S1). (**B**,**C**) Genomic organization and synteny comparisons among human *GFRA1*, rodents *Gfrα1* and zebrafish *gfrα1b* (**B**) or zebrafish *gfrα1a* (**C**). The syntenic regions were analyzed according to the alignment of the target genes and genomic annotation available in the GenBank database (National Center for Biotechnology Information and Ensembl).

Phylogenetic analysis further confirmed that both zebrafish Gfrα1a and Gfrα1b are related to other fish Gfrα1a and Gfrα1b predicted sequences, respectively, and that these isoforms diverge and form two separate fish-specific subclades (estimated posterior probability = 1) (Figure 2A). On the other hand, the GFRA1 sequences from other vertebrates (mammals, birds, reptiles, amphibians and Chondrichthyes) are clustered and form a separate clade to the fish Gfrα1 (estimated posterior probability = 0.851) (Figure 2A).

A cross-species comparison of chromosome neighboring genes revealed that both the zebrafish *gfrα1a*- and *gfrα1b*- containing regions are syntenic to human *GFRA1*- and rodent *Gfrα1*-containing regions (Figure 2B). This analysis also showed that the zebrafish *gfrα1b* gene (chromosome 12, NC_007123.7) showed a larger group of syntenic genes (8 out of 14 genes analyzed) when compared with zebrafish *gfrα1a* (chromosome 13, NC_007124.7) (2 out of 14 genes analyzed) (Figure 2C).

### 3.2. Expression Profiling in Zebrafish Testes and Identification of Gdnfa-, Gfrα1a- and Gfrα1b-Expressing Cells

**Figure 3:**
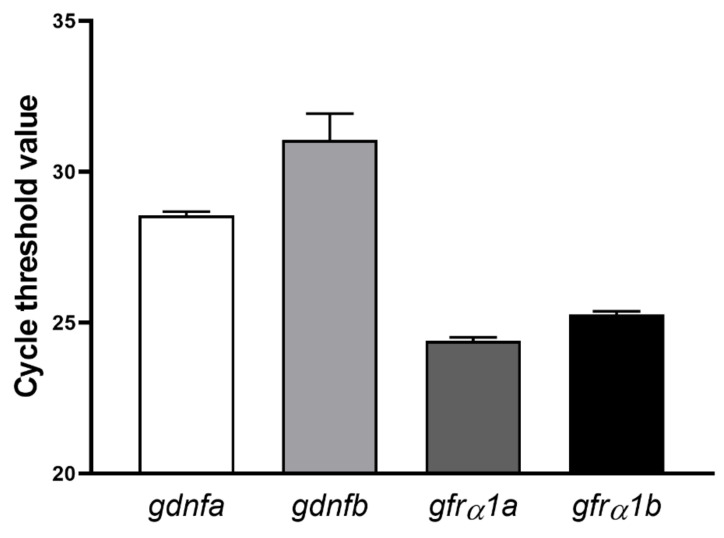
Number of amplification cycles (cycle threshold (Ct)) for both ligands (*gdnfa* and *gdnfb*) and receptors (*grfα1a* and *grfα1b*) in zebrafish testes. Bars represent the mean ± SEM (*n* = 4) for each transcript.

RT-qPCR analyses revealed that both ligands (*gdnfa* and *gdnfb*) and receptors (*gfrα1a* and *gfrα1b*) were expressed in zebrafish testes, although with different numbers of amplification cycles (i.e., values of cycle threshold (Ct)) (Figure 3). As the Ct for *gdnfb* is greater than 30, this value indicates lower amounts for this target nucleic acid in zebrafish testes (Figure 3).

**Figure 4:**
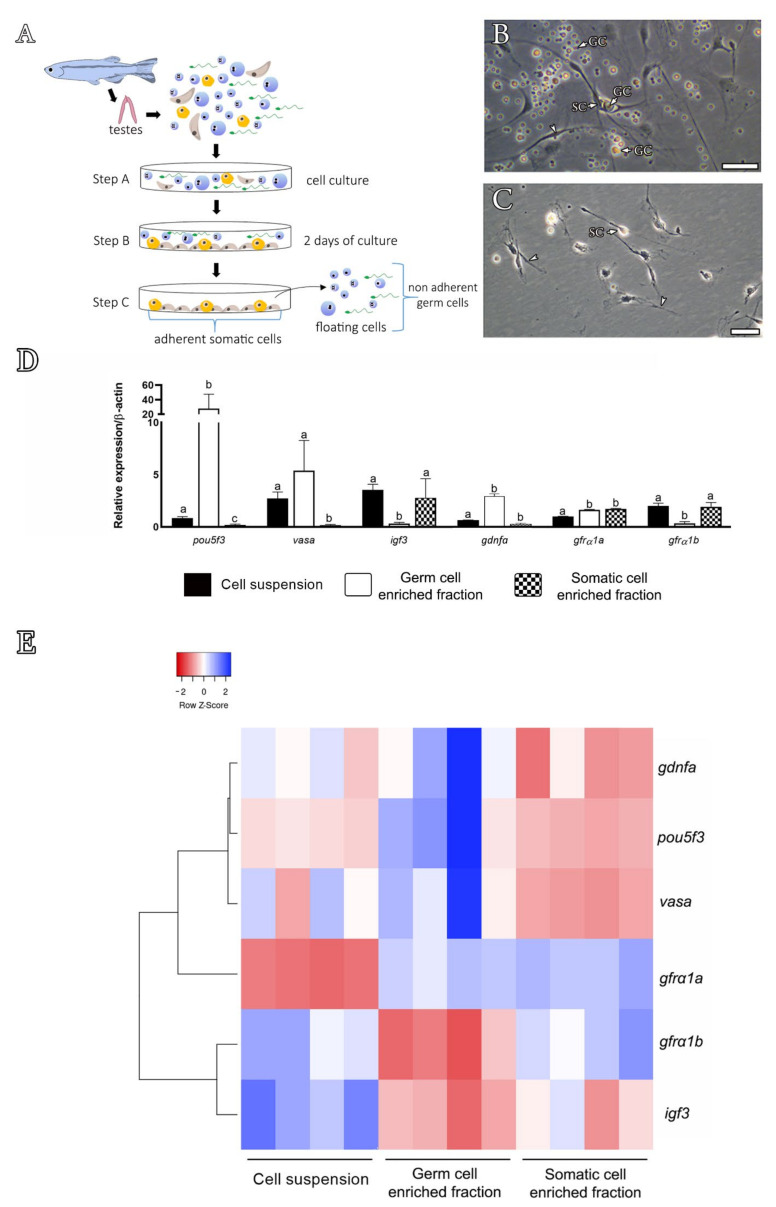
Differential plating method and expression analysis of the cellular enriched fractions. (**A**) Scheme showing the steps of the differential plating method, according to Hinfray et al. [51]. Briefly, a total testicular cell suspension was harvested (step A) in L-15 culture medium, and after 2 days of culture, only somatic cells (Sertoli cells, brown triangular shapes; Leydig cells, yellow oval shapes) adhere to the bottom of the plate (step B), while germ cells (blue shapes) remain floating or loosely attached to the bottom of the plate (step C). After washing steps, germ cells (floating and weakly attached to the somatic cells) can be removed, leaving the adherent somatic cells at the bottom of the plate. The firmly attached somatic cells can be obtained after extensive washing with trypsin. (**B**) Total testicular cell suspension after 2 days of culture. Note the somatic adherent cells (SCs) with cytoplasm extensions towards different germ cells (GC). (**C**) After washing, note that only somatic adherent cells (SCs) remain attached to the bottom of the plate. Scale bars: 20 µm. (**D**,**E**) Gene expression analysis of isolated zebrafish testicular cell populations: total cell suspension (black bar), germ cell-enriched population (white bar) and testicular somatic cells (hatched bar). Cells were obtained from three independent experiments. Bars represent relative mRNA levels of target genes expressed as mean ± SEM; different letters indicate significant differences between the cell populations (one-way ANOVA followed by the Student–Newman–Keuls test). (**E**) Heat map illustrating the relative mRNA levels of *pou5f3, vasa, igf3 gdnfa, gfrα1a* and *gfrα1b* according to different cell populations. Data shown are log2 values (relative quantification) relative to the average expression. Each colored cell in the heat map represents the standardized relative gene expression value for each sample. Genes (rows) are hierarchically clustered using Pearson correlation and the distance metric. The higher expression values are displayed in blue, moderate expression values in shades of white (light blue and light red) and lower expression values in red.

Considering the lower amounts of *gdnfb* transcripts in zebrafish testes, we focused our analysis on *gdnfa.* We tried to identify the cellular types expressing *gdnfa* mRNA in zebrafish testes by employing in situ hybridization with a specific antisense cRNA probe (Table 1, Figure S1) and RT-qPCR using RNA from isolated testicular cell populations (germ and somatic cell-enriched populations) (Figure 4). The first approach showed that *gdnfa* is expressed in germ cells (Figure S1). Nevertheless, due to limited resolution, it was not possible to unravel whether the signal was present or not in the Sertoli cells (Figure S1). This was attributed to the fact that cytoplasmic extensions of Sertoli cells protrude towards the lumen of a cyst in between the germ cells, making it difficult to accurately locate the signal. The precise identification of *gdnfa* expression sites was then accomplished through RT-qPCR using testicular cell populations obtained after the differential plating method (Figure 4A–E). In this approach, expression analysis showed higher transcript levels for *gdnfa* in the germ cell-enriched population when compared to the levels found in the total testicular cell suspension (Figure 4D,E). When analyzing the testicular somatic cell population, we found that *gdnfa* mRNA levels decreased significantly as compared to the levels observed in the germ cell fraction (Figure 4D,E). To confirm this result, we performed proper controls using specific markers for germ (*vasa* and *pou5f3*) and Sertoli cells (*igf3*). For the germ cells, we used *vasa*, which is a germ cell marker mostly expressed in early germ cells, including types A_und_, A_diff_ and B spermatogonia [47]. We showed that *vasa* was expressed in the germ cell-enriched population, although with levels not significantly higher as compared to the total cell suspension (Figure 4D,E). On the other hand, *vasa* was not expressed in the testicular somatic cell fraction (Figure 4D,E). For *pou5f3*, a marker of types A_und_, A_diff_ and B spermatogonia (Souza, Doretto and Nóbrega (unpublished data)), we showed higher mRNA levels in the germ cell-enriched fraction, but no expression in the somatic cell population (Figure 4D,E). For the Sertoli cells, we used *igf3*, which is a growth factor produced by Sertoli cells [54]. *igf3* was not expressed in the germ cell population but it was detected in the somatic cell fraction with levels comparable to those found in the total cell suspension (Figure 4D,E).

We also expressed our data in a heat map and genes were hierarchically clustered using Pearson correlation and the distance metric (Figure 4E). We showed through this analysis that genes such as *vasa*, *pou5f3* and *gdnfa* were hierarchically clustered in the germ cell fraction and separated from *igf3* and *gfrα1b*, which were clustered in the somatic cell fraction (Figure 4E). *gfrα1a* was expressed in both germ and somatic cell fractions (Figure 4D,E).

### 3.3. Localization of Gfrα1a Protein in Zebrafish Testis

**Figure 5:**
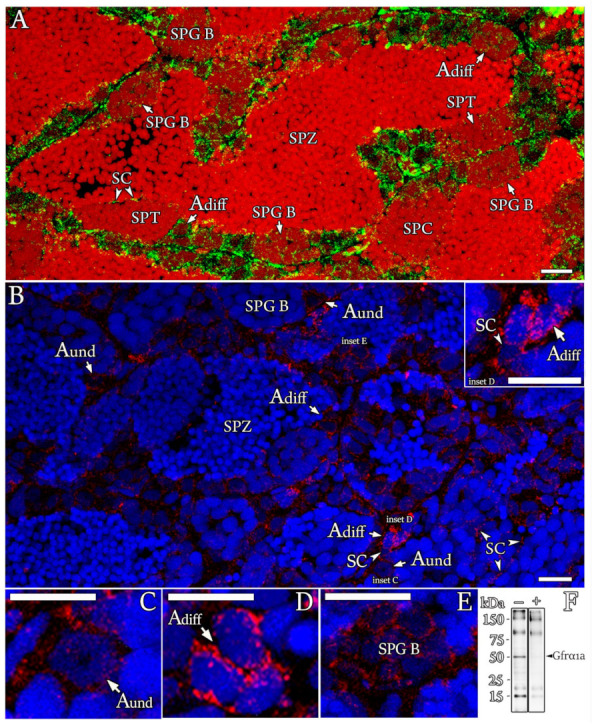
Cellular localization of Gfrα1a in zebrafish testis. (**A**–**E**) Immunofluorescence for Gfrα1a (green—**A**; red—**B**–**E**) in testis sections of sexually mature zebrafish. The spermatogonial generations, including type A undifferentiated spermatogonia (A_und_), type A differentiated spermatogonia (A_diff_) and type B spermatogonia (SPG B), were immunoreactive to Gfra1a, although staining patterns among them varied according to developmental stage. The signal was not found in spermatocytes (SPCs), spermatids (SPTs) and spermatozoa (SPZ). Note that Sertoli cells (SCs) contacting germ cells at different stages of development were also immunoreactive to Gfrα1a. Cell nuclei were counterstained with propidium iodide (**A**) or Hoechst (**B**–**E**). Scale bars: 15 µm. (**F**) Gfrα1a (approximately 52 kDa (kilodaltons)) immunoblots of whole testes with (+) or without (−) preadsorbed antibodies, confirming the presence of the protein in the zebrafish testes and antibody specificity.

Gfrα1a was detected in all generations of zebrafish spermatogonia, although the staining pattern varied among them according to the developmental stage (Figure 5A,C–E). The Gfrα1a signal was finely dispersed in the cell surface and cytoplasm of type A_und_ spermatogonia (Figure 5C) and later became more aggregated, forming intensely stained spots in type A_diff_ spermatogonia (Figure 5D). In type B spermatogonia, the Gfrα1a signal became finely dispersed again (Figure 5E) and gradually decreased as the number of spermatogonia B increased within the cyst until it became undetectable in the meiotic and post-meiotic cysts (Figure 5A). Furthermore, Gfrα1a was also found in Sertoli cells contacting germ cells at different stages of development (Figure 5A,B (inset) and Figure S1F,G). This result was also confirmed by the expression of both *gfrα1a* and *gfrα1b* in the somatic cell-enriched population (Figure 4D). Altogether, these two bodies of evidence support the presence of Gfrα1a and 1b in zebrafish Sertoli cells. The specificity of the antibody (anti-zebrafish Gfrα1a) was confirmed by immunoblots (Figure 5F) and control sections either by using a preadsorbed antibody with the corresponding peptide or omitting the primary antibody (Figure S2). It is important to mention that the immunofluorescence signal should not be limited to Gfrα1a, since the antibody could potentially recognize part of zebrafish Gfrα1b (see the blue line in Figure 1). 

### 3.4. Three-Dimensional Model for Predicting the Interaction between rhGDNF and Zebrafish Gfrα1a

**Figure 6:**
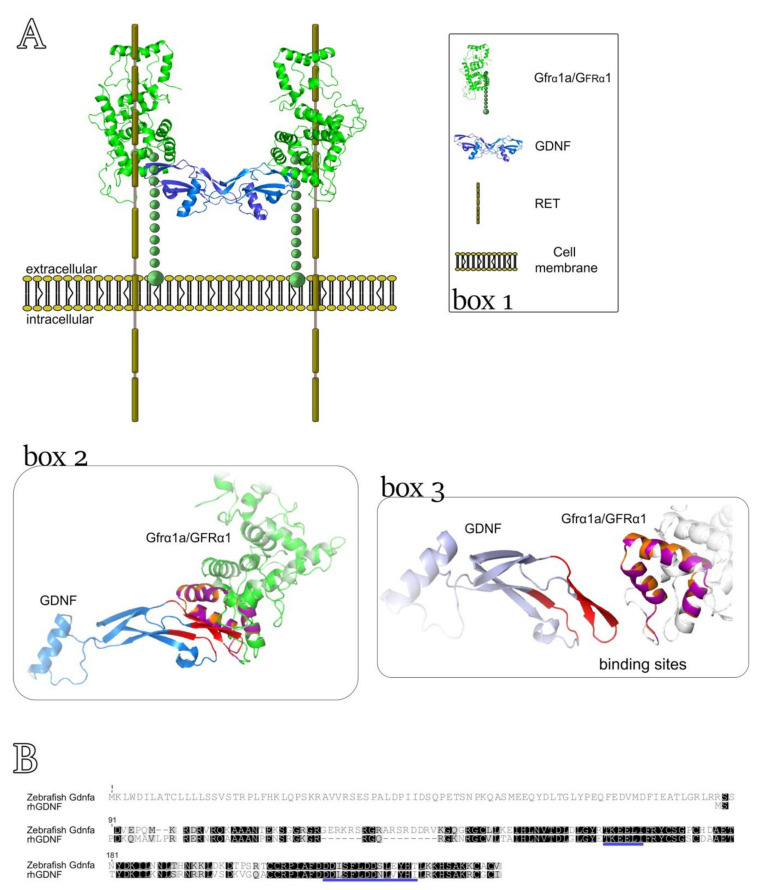
A 3D model to predict the interaction between rhGDNF and zebrafish Gfrα1a. (**A**) Box 1 depicts the molecular components of the complex GDNF-GFRα1-RET. Boxes 2 and 3 show the predictive 3D model (template 4ux8.1) in which the structural similarities between zebrafish Gfrα1a and human GFRA1 are represented by orange and purple coloring and the identity of the structure formed at the binding sites is indicated in red. In box 2, green is used to indicate the conserved amino acid sequences between zebrafish Gfrα1a and human GFRA1 and blue indicates the GNDF protein. In box 3, we highlighted the interaction sites between human GDNF and zebrafish Gfrα1a/human GFRA1. (**B**) Alignment of zebrafish Gdnfa with rhGDNF. The blue lines indicate the conserved binding sites to zebrafish Gfrα1a or human GFRA1.

In this study, we used a recombinant human hormone because the recombinant zebrafish Gdnf is not commercially available. Therefore, to investigate whether rhGDNF could have effects on zebrafish spermatogenesis, we first generated a 3D structure model to predict the possible interaction sites between human GDNF and zebrafish Gfrα1a (Figure 6A, box 2, box 3). The 3D structure (hetero-2-2-mer) was built according to the homology of the 4ux8.1 template and showed a GMQE value of 0.63 with 74% of identity and a resolution of 24Å (method: Electron Microscopy) when compared to human GDNF-GFRA1 interaction (merged in the 3D structure) (Figure 6A, box 2, box 3). Moreover, the predictive model demonstrated that 89.8% of the amino acid residues were in the most favorable regions, 7% of residues were situated in allowed regions (~2% expected) and 3.1% in the outlier regions according to Ramachandran plots. The 3D structures of the hetero-2-2-mer (GDNF-zebrafish Gfrα1a) were based on the homology modeling templates and are shown in Figure 6A (box 2, box 3). More detailed information regarding the predictive interaction model between GDNF and zebrafish Gfrα1a can be found in the Supplementary Materials (Figure S3, Video S1). In agreement with the 3D model, the alignment of zebrafish Gdnfa with rhGDNF showed conserved regions, particularly in the binding sites to human GFRA1 or zebrafish Gfrα1a (Figure 6B).

### 3.5. Biological Effects of rhGDNF

**Figure 7:**
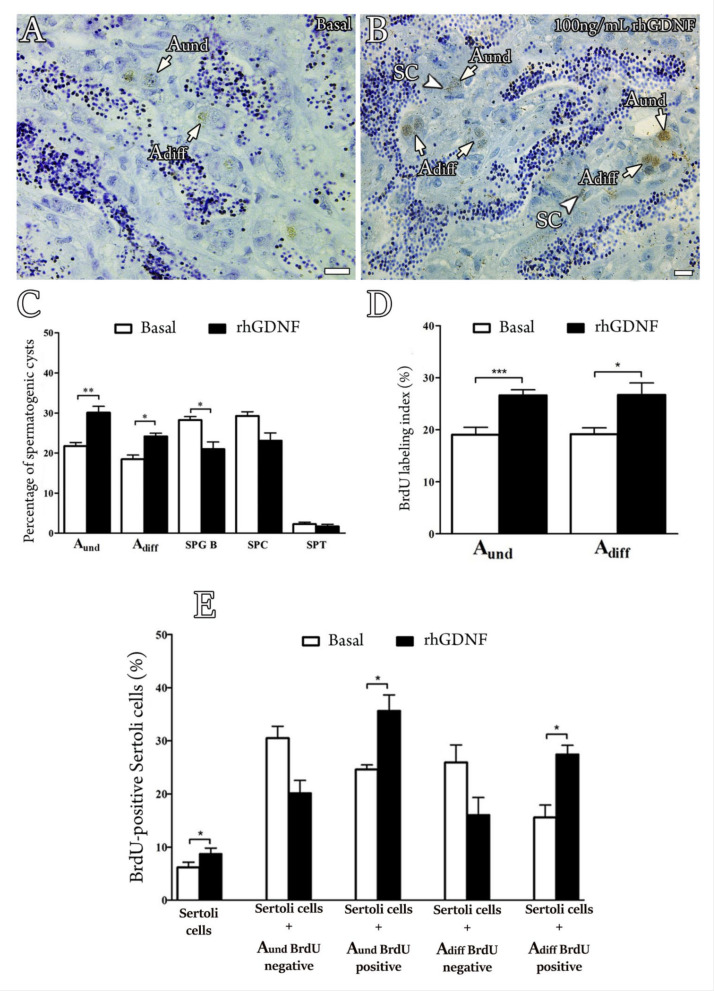
Effects of Gdnf on germ cell composition and cellular proliferation, using a previously established primary testis tissue culture system. (**A**,**B**) BrdU immunodetection from zebrafish testicular explants incubated for 7 days in the absence (Basal) or presence of rhGDNF (100 ng/mL), demonstrating a higher proliferation activity for type A undifferentiated spermatogonia (A_und_) and type A differentiated spermatogonia (A_diff_) in the presence of rhGDNF. (**C**) Frequency of different germ cell cysts after 7 days of incubation in the absence (Basal) or presence of rhGDNF (100 ng/mL). Types A_und_, A_diff_ and B spermatogonia (SPG B), spermatocytes (SPCs) and spermatids (SPTs) were identified according to morphological characteristics, as described by Leal and collaborators [55]. (**D**) Mitotic indices of type A_und_ and A_diff_ spermatogonia after incubation in the absence (Basal) or presence of rhGDNF (100 ng/mL) for 7 days. (**E**) Mitotic indices of Sertoli cells in association with BrdU-negative or BrdU-positive type A_und_ and A_diff_ spermatogonia in the absence (Basal) or presence of rhGDNF (100 ng/mL) for 7 days. Sertoli cells were identified according to morphological characteristics, as described previously [55]. In fish, Sertoli cells (SCs) have a triangular nuclear shape, dark chromatin and usually they appear surrounding spermatogenic cysts, as shown in Figure S1. Bars represent the mean ± SEM (*n* = 10). Paired *t*-test, * *p* < 0.05, ** *p* < 0.01, *** *p* < 0.001. Scale bars: 15 µm.

To investigate the roles of Gdnf in zebrafish spermatogenesis, we first examined whether rhGDNF could affect germ cell composition and cellular proliferation, using a previously established primary testis tissue culture system (Figure 7A–D). The results showed that rhGDNF (100 ng/mL) increased the abundance of types A_und_ and A_diff_ spermatogonia as compared to basal conditions (Figure 7C). These data are also consistent with the proliferation activity of these cells, showing that treatment with rhGDNF (100 ng/mL) augmented the mitotic index of both types of spermatogonia (A_und_ and A_diff_) as compared to their basal mitotic index (approximately 1,5-fold increase for A_und_ and A_diff_, with *p* < 0.001 and *p* < 0.01, respectively) (Figure 7A,B,D). Moreover, histomorphometrical analysis showed that rhGDNF decreased the frequency of type B spermatogonia, whereas no effects were observed for meiotic and post-meiotic germ cells (Figure 7C). In this study, we also quantified Sertoli cell proliferation (Figure 7E), reasoning that change in the proliferation of Sertoli cells associated with types A_und_ or A_diff_ spermatogonia would indicate the creation of new niche space or support the development of differentiating spermatogonial cysts, respectively [62]. Our results then demonstrated that treatment with rhGDNF stimulated Sertoli cell proliferation (1,5-fold increase, *p* < 0.050), particularly if the Sertoli cells associated with proliferating types A_und_ and A_diff_ spermatogonia (Figure 7E).

**Figure 8:**
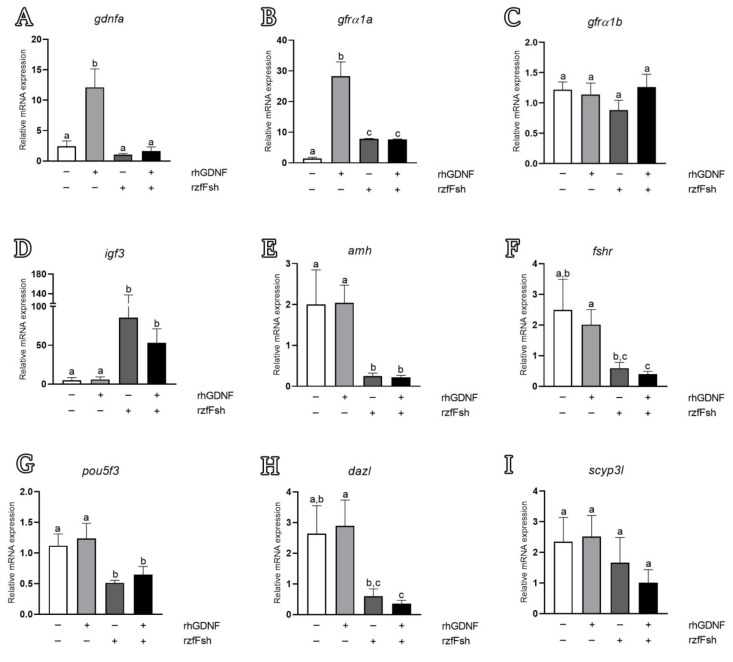
Relative mRNA levels of genes related to Gdnf signaling (*gdnfa*, *gfrα1a* and *gfrα1b*) (**A–C**), Sertoli cell growth factors (*igf3* and *amh*) (**D–E**), Fsh signaling (*fshr*) (**F**) and germ cell markers (undifferentiated spermatogonia—*pou5f3* (**G**); differentiated spermatogonia and preleptotene spermatocytes—*dazl* (**H**); and primary spermatocytes—*scyp3l (***I***)*). Testicular explants were cultivated for 7 days with rhGDNF, rzfFsh or both (rhGDNF + rzfFsh). The relative mRNA levels were normalized with the *β-actin* levels. Bars represent the mean ± SEM (*n* = 20). One-way ANOVA followed by the Student–Newman–Keuls test, in which different letters denote significant differences (*p* < 0.05) among treatment conditions.

In order to elucidate the molecular mechanisms mediated by rhGDNF on basal or Fsh-induced spermatogenesis, we performed gene expression analyses of selected genes related to Gdnf signaling (*gdnfa*, *gfrα1a* and *gfrα1b*), Sertoli cell growth factors (*igf3* and *amh*), Fsh signaling (*fshr*) and germ cell markers (undifferentiated spermatogonia—*pou5f3*; differentiated spermatogonia and preleptotene spermatocytes—*dazl;* and primary spermatocytes—*scyp3l*) (Figure 8).

RT-qPCR analysis revealed that rhGDNF increased the transcript levels of *gdnfa* and *gfrα1a*, whereas *gfrα1b* mRNA levels remained unaltered when compared with basal condition levels (Figure 8A–C). The transcript abundance for the other genes (Sertoli cell growth factors, Fsh signaling and germ cell markers) did not change following rhGDNF treatment (Figure 8D–I). We further investigated whether rhGDNF could affect the Fsh-induced changes in testicular gene expression, since Fsh is considered the major endocrine player regulating the zebrafish spermatogonial phase [48,54,63]. We first showed that Fsh did not modulate the transcript levels of *gdnfa*, *gfrα1a* or *gfrα1b* in the zebrafish testes (Figure 8A–C). However, Fsh was able to nullify the rhGDNF-increased *gdnfa* and *gfrα1a* mRNA levels following co-treatment (Figure 8A, B). With respect to Sertoli cell growth factors, we demonstrated that rhGDNF did not change Fsh-mediated expression on *igf3* (Figure 8D) or *amh* mRNA levels (Figure 8E). As expected, and in agreement with previous studies [54,64], Fsh increased *igf3* mRNA levels (Figure 8D) and down-regulated *amh* transcription (Figure 8E). The other evaluated genes were not responsive to Fsh or co-treatment (Figure 8F–I). Nevertheless, it is worth mentioning that transcript levels of *fshr*, *pou5f3* and *dazl* were significantly higher following rhGDNF treatment than in the co-treatment with Fsh (Figure 8F–H).

### 3.6. In Silico Analysis of Putative Regulatory Sequences Upstream of Human GDNF, Mouse Gdnf and Zebrafish Gdnfa

**Figure 9:**
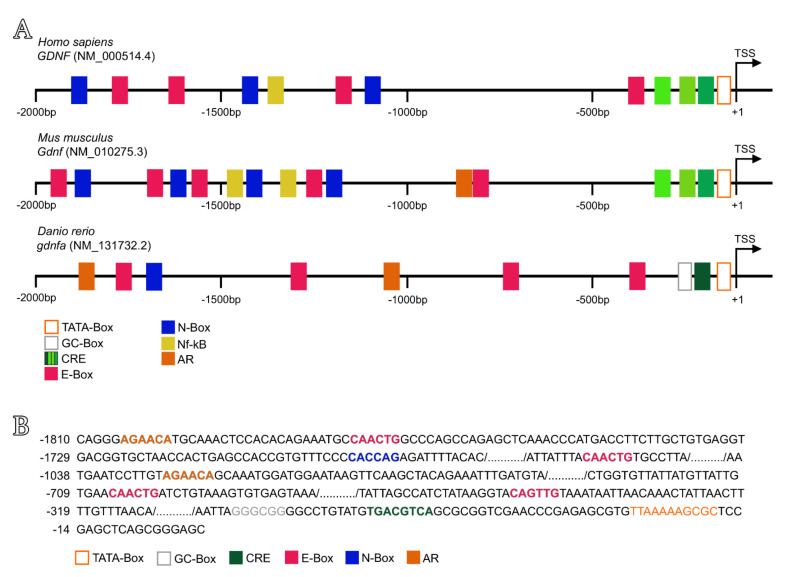
Predicted regulatory sequences upstream of human *GDNF*, mouse *Gdnf* and zebrafish *gdnfa*. (**A**) The upstream region (2000 bp) of human *GDNF* contains three different sequences of cAMP response elements (CRE), four E-box sequences, three N-box sequences and one Nf-kB binding site. The upstream region (2000 bp) of mouse *Gdnf* contains an N-box/E-box-rich region at bp −1300 to −1900 and additional E-boxes downstream, one androgen receptor binding site (AR), two Nf-kB binding sites and three different sequences of CRE close to the TSS (transcriptional start site). The upstream region (2000 bp) of zebrafish *gdnfa* contains four E-box sequences and one N-box sequence, two AR half sequences and only one CRE close to a GC-box and the TSS. TSS is the transcription start site (position +1). (**B**) Sequences of putative binding sites upstream of zebrafish *gdnfa*. In the open orange box is shown the TATA-box sequence, in the open gray box the GC-box, in the dark green box the CRE, in the pink box the E-box, in the blue box the N-box, and in the filled orange box the AR half binding site.

To support our expression analysis, we investigated the putative regulatory sequences upstream of the transcriptional start site (TSS) of human *GDNF* (NM_000514.4), mouse *Gdnf* (NM_010275.3) and zebrafish *gdnfa* (NM_131732.2) (Figure 9). The in silico analysis showed three different types of cAMP response elements (CRE), several N-box and E-box motifs, one NF-kB binding site and a TATA-Box within the 2000 bp upstream of human GDNF (Figure 9, Table S2). The upstream sequence of the *Gdnf* mouse gene showed similar regulatory binding sites to the human *GDNF* (Figure 9, Table S2). For zebrafish, we predicted a non-canonical TATA-Box, one CRE close to a GC-Box, one N-Box, four E-Boxes and two androgen receptor (AR) half binding sites within the 2000 bp upstream of *gdnfa* (Figure 9, Table S2).

## 4. Discussion

This study demonstrated the involvement of the Gdnf/Gfrα1 signaling pathway in the regulation of the spermatogonial phase in zebrafish. Our first analysis identified two zebrafish paralogs for the Gfrα1-encoding gene, named zebrafish *gfrα1a* and *gfrα1b*. The predicted amino acid sequences of zebrafish Gfrα1a and Gfrα1b revealed high identity to GFRα1 from other mammalian species investigated in this study (>60% and >57% sequence identity for Gfrα1a and Gfrα1b, respectively). Moreover, both paralogs have conserved domains and residues which are typical of GFRα1 family members, such as 3 cysteine-rich domains (D1, D2 and D3), 28 cysteine residues (plus 2 in the terminal region) and 2 triplets (MLF and RRR) [65,66,67]. Studies in mice using site-directed mutagenesis have shown that some of these conserved regions (e.g., two triplets—MLF and RRR—in the D2 domain) are critical for Gfrα1 binding to Gdnf, activation of the receptor complex and elicitation of downstream signal transduction [65,67]. This evidence suggested that, theoretically, both zebrafish Gfrα1a and Gfrα1b could bind and elicit a response to Gdnf/GDNF (e.g., rhGDNF). Moreover, in agreement with previous studies [33,68], phylogenetic analysis demonstrated that zebrafish Gfrα1a and 1b are clustered with other fish Gfrα1a and 1b sequences; the paralogs diverged, forming two distinct sub-clades within the fish clade. Additional analysis of chromosome neighboring genes revealed that both zebrafish *gfrα1a*- and *gfrα1b*-containing regions are syntenic to human *GFRA1*- and rodent *Gfrα1*-containing regions. Altogether, this evidence confirmed that zebrafish *gfrα1a* and *gfrα1b* are duplicated genes that diverged from each other after the teleost-specific whole genome duplication. It is well established that, around 320 million years ago, the common ancestor of the teleosts experienced a third round of whole genome duplication [69,70]. This event was responsible for the generation of a large number of duplicated genes that could follow different evolutionary paths, such as co-expression (both copies retain the ancestral function), non-functionalization (function loss or complete deletion of one copy), sub-functionalization (specialization of each copy, sub-function partition), or neo-functionalization (acquisition of a novel function) [69,70]. In this study, we could not determine the specific roles of Gfrα1a and Gfrα1b. Additional studies (e.g., specific knockouts of each copy) are required to confirm this hypothesis and to unravel the specific roles for each Gfra1 paralog in zebrafish spermatogenesis.

When evaluating the expression profiling of Gfrα1a and Gfrα1b, we found that both paralogs are expressed in zebrafish testes. Considering the greater homology with the mammalian GFRA1 and the modulation by rhGDNF, we developed an antibody for zebrafish Gfrα1a (although it could be able to recognize zebrafish Gfrα1b). Our data revealed that Gfrα1a was found in all types of zebrafish spermatogonia, although the staining pattern varied among the different generations of spermatogonia. Gfr*α*1a was mainly detected in early types of spermatogonia (A_und_ and A_diff_), and immunostaining decreased as spermatogonial clones became larger and more differentiated. Likewise, accumulating evidence has shown that GFRA1 is a conserved marker for mammalian type A undifferentiated spermatogonia [22,23,24,25,26,27,28,71,72,73] and the frequency of GFRA1+ spermatogonia decreases as spermatogonia progress from A_s_ to Aal [72,73]. Similarly, in other fish species, mRNA or protein levels of Gfrα1a were found mainly in type A_und_ spermatogonia of dogfish (*Scyliorhinus canicula*) [29,53], rainbow trout (*Oncorhynchus mykiss*) [30,31,32], medaka (*Oryzias latipes*) [33] and tilapia (*Oreochromis niloticus*) [73]. In rainbow trout, Nakajima et al. [30] reported that *gfrα1* transcripts decreased throughout spermatogonial development and became undetectable in spermatids and spermatozoa. In medaka, Zhao et al. [33] showed a moderate signal for *gfrα1a* and *gfrα1b* mRNA in spermatocytes, but no expression was found in spermatids and spermatozoa. Altogether this evidence is in agreement with our results and supports our hypothesis that the Gndf-Gfr*α*1a signaling pathway is important for the regulation of the zebrafish spermatogonial phase but is not required for meiotic and post-meiotic phases. Strikingly, our study also detected the Gfrα1a protein among Sertoli cells associated with different types of germ cells. In rainbow trout, Maouche et al. [68] demonstrated that *gfrα1a1* transcripts were mainly expressed in somatic testicular cells, while *gfrα1a2* was restricted to type A_und_ spermatogonia. To our knowledge, our study and the one of rainbow trout [68] were the first to show that Gndf-Gfr*α*1a is not only involved in the control of type A_und_ spermatogonia but can also modulate the functions of Sertoli cells.

Investigation of the Gdnf ligands (Gdnfa and Gdnfb) revealed that both are expressed in zebrafish testes, although *gdnfb* has shown a Ct value greater than 30. These data suggest that Gdnfa might be the main ligand in zebrafish testes. Further in situ hybridization and RT-qPCR analysis demonstrated that *gdnfa* is mainly expressed in the germ cells. *gdnfa* was not expressed in somatic testicular cells. In both analyses, we were not able to identify the germ cell types expressing *gdnfa* in zebrafish testes. Nakajima and collaborators [30], on the other hand, demonstrated that *gdnf* mRNA and protein were expressed in type A_und_ spermatogonia of immature rainbow trout. Moreover, the same authors showed that *gdnf* and *gfrα1* were co-expressed and that their expression changed synchronously during germ cell development [30]. Altogether, this evidence supports our findings that zebrafish Gdnfa is a germ cell-derived factor that exerts autocrine and paracrine functions on spermatogonia and Sertoli cells, respectively, in zebrafish testes. Moreover, these data provide new insights into the Gndf-Gfrα1a signaling pathway in fish as compared to mammals. In mammals, GDNF is secreted by testicular somatic cells (Sertoli cells [2,9,10], peritubular myoid cells [11,12] and testicular endothelial cells [14]), acting only as a paracrine factor for GFRA1-expressing undifferentiated spermatogonia [21,22,23,24,25,26,27,28,71,72]. This difference is likely related to the events that took place after the teleost-specific whole genome duplication, involving, for example, non- and neo-functionalization of the Gdnf paralogs. Moreover, these findings suggest that the common vertebrate ancestor expressed Gdnf in testicular somatic cells, while Gdnf expression in germ cells is considered an evolutionary novelty which is exclusive to fish.

To assess the biological roles of Gdnf in zebrafish spermatogenesis, we used a rhGDNF. There is strong evidence that rhGDNF can bind to zebrafish Gfrα1a and elicit a downstream signal transduction in zebrafish testes. The first item of evidence is the predictive 3D model which examined the interaction sites between human GDNF and zebrafish Gfrα1a based on the binding interaction with human GFRA1. This analysis revealed structural similarities between zebrafish Gfrα1a and human GFRA1 (Figure 6A, box 2), and higher identity of the structure formed at the binding sites between human GDNF and human GFRA1, and with Gfrα1a zebrafish (Figure 6A, box 2). Moreover, this analysis also showed that most of the amino acid residues identified as crucial for ligand–receptor interactions are conserved in the zebrafish Gfrα1a, with exceptions for the residues Gly155 and Ile175, which were replaced by Glu and Thr, respectively. The predictive 3D model was also supported by Ramachandran plots which showed that 89.8% of the amino acid residues were in the most favorable regions, 7% of residues situated in allowed regions (~2% expected) and 3.1% in outlier regions. The second item of evidence is the sequence alignment demonstrating conserved regions between rhGDNF and zebrafish Gdnfa, such as the binding sites to GFRA1/Gfrα1a. The last item of evidence is the capability of rhGDNF to induce proliferation and modulate gene expression in zebrafish testes (see below), indicating that rhGDNF not only can bind to zebrafish Gfrα1a but also can trans-activate the receptor complex and trigger molecular and cellular responses.

With regard to biological functions, our results demonstrated that rhGDNF (100 ng/mL) increased the mitotic index of types A_und_ and A_diff_ spermatogonia when compared to basal conditions. Consistently, histomorphometric analysis revealed that both types A_und_ and A_diff_ became more abundant, while type B significantly decreased following rhGDNF treatment. Altogether, these results indicated not only that Gdnf stimulates proliferation of the most undifferentiated spermatogonia (A_und_ and A_diff_) but that it is also involved in blocking late differentiation into type B spermatogonia. Similar functions have been described in mammalian and non-mammalian species. In mammalian species, particularly rodents, GDNF promotes self-renewing proliferation of SSCs ([2]; see reviews in Parekh et al. [7] and Mäkelä and Hobbs [8]), although a recent study in mice has shown that GDNF could be more associated with blocking differentiation rather than actively stimulating SSC proliferation [4]. In dogfish, rhGDNF promoted in vitro proliferation and long-term maintenance of spermatogonia with stem characteristics [53]. In medaka, Wei et al. [34] demonstrated that recombinant medaka Gdnfa and Gdnfb were involved in the proliferation and survival of medaka SSCs. Furthermore, the knockdown of medaka *gfrα1a* and *gfrα1b* subsequently confirmed that both receptors mediated the proliferation and survival of medaka SSCs [33]. In this study, Zhao et al. [33] also showed that genes related to differentiation (e.g., *c-kit*) were up-regulated when the expression of both receptors was lowered. Altogether, this evidence from different species sustains our conclusion that the Gndf-Gfr*α*1 signaling pathway is associated with maintaining the pool of undifferentiated spermatogonia (A_und_ and A_diff_) through promoting their proliferation and also by inhibiting their differentiation. Moreover, as zebrafish Gdnfa and its receptor (Gfrα1a) are co-expressed, it is important to highlight that the above-mentioned function is an autocrine loop of Gdnf on types A_und_ and A_diff_ spermatogonia.

In this study, we also quantified Sertoli cell proliferation because change in the proliferation of Sertoli cells associated with types A_und_ or A_diff_ spermatogonia would indicate the creation of new niche space or support for the development of spermatogonial cysts, respectively [62]. In fish, in contrast to mammals, Sertoli cells are not terminally differentiated and continue to proliferate during spermatogenesis in adult males of different species, including zebrafish [52,71,74]. Strikingly, our results demonstrated that Gndf promotes proliferation of Sertoli cells that are particularly associated with types A_und_ and A_diff_ spermatogonia which are also undergoing mitosis (BrdU-positive cells). These data indicate for the first time that a germ cell-derived factor is involved in the creation of new spermatogenic cysts, i.e., new available niches, in addition to supporting the development of early differentiating spermatogonial cysts. In the first case, as Gdnf stimulates the proliferation of type A_und_, the newly formed, single spermatogonium must recruit its own Sertoli cells to form a new spermatogenic cyst. Therefore, it is reasonable that new Sertoli cells would be produced to create a niche into which the newly formed, single type A_und_ can be recruited or attracted (germ cell homing). Consistently, in mice, Gdnf has been shown to be important for germ stem cell homing as it acts as a SSC chemotactic factor [75]. In the second case (supporting the development of differentiating spermatogonial cysts), Gdnf-induced Sertoli proliferation would provide structural and nutritional support for the development of early differentiating spermatogonia. In both cases, Gdnf effects on Sertoli cells might be mediated directly through Gfr*α*1a, which is also expressed in Sertoli cells of zebrafish. In agreement with our observation, a study in rodents has shown that Gdnf promoted the proliferation of immature Sertoli cells through its interaction with Gfrα1 and neural cell adhesion molecules (NCAMs), both co-expressed in Sertoli cells [76,77]. Although there is evidence of Gfr*α*1a expression in Sertoli cells, we cannot exclude that Gdnf-induced Sertoli cell proliferation may be mediated by other growth factor(s) produced by type A undifferentiated spermatogonia.

We further evaluated whether Gdnf could modulate testicular gene expression or affect Fsh-induced gene expression in zebrafish explants. Previous studies have shown that Fsh is the major endocrine player regulating zebrafish spermatogonial development through targeting Sertoli and Leydig cell functions, such as sex steroid and growth factor production [47,54,63,78,79]. Our results showed that Gdnf positively modulates its own regulatory pathway (Gdnfa-Gfr*α*1a). This would be the first demonstration that a germ cell factor can affect the spermatogonial niche through an autocrine and paracrine loop. It seems that Gdnf signaling would enhance its own production and sensitivity to favor the creation of new spermatogonial niches (type A_und_ spermatogonia and Sertoli cells). Notably, *gfrα1b* was not modulated by any treatment, which indicates that zebrafish Gfrα1a may be the mammalian GFRα1 homologous form. Moreover, we showed that Fsh did not modulate *gdnfa* expression in zebrafish testis explants. Similarly, Bellaiche et al. [31] demonstrated that Fsh did not modulate the expression of *gdnfb* in immature and early maturing rainbow trout testicular explants either. This regulation in fish is different from the one reported in mammals, where Fsh has been shown to stimulate the expression of *Gdnf* in the testes [79]. One possible explanation for this different regulation would be the distinct cellular sites expressing Gdnf in mammalian and fish testes. In zebrafish, Gdnf is mainly secreted by germ cells, which are not the direct targets of Fsh, while in mammals, Gdnf is secreted by somatic cells, including Sertoli cells, which are known to express Fsh receptors. Additionally, to support our data, we performed an in silico analysis within regions −2000 to +1 bp upstream of the zebrafish *gdnfa* gene to search cAMP response elements (CREs). As is well known, Fsh stimulates the cAMP-dependent protein kinase A signaling pathway, leading to phosphorylation of the cAMP response element-binding protein (CREB), which is necessary to transactivate several genes containing CREs [80,81]. Lamberti and Vicini [59] demonstrated that three CRE binding sites in the murine *Gdnf* promoter are directly involved in basal and cAMP-induced expression of *Gdnf* in Sertoli cells. In our in silico analysis, we demonstrated that the zebrafish *gdnfa* promoter (−2000 to +1 bp) has fewer conserved DNA binding sites compared with human and mouse *GDNF*/*Gdnf* promoters. Moreover, our analysis showed only one CRE site near to the zebrafish *gdnfa* transcription start site, instead of three CREs, as reported in human and mouse. The difference in the promoter region and the lower number of CRE binding sites could be the reason that Fsh could not stimulate *gdnfa* expression in zebrafish testes.

The *GDNF*/*Gdnf* promoter region also contains several E- and N-boxes that allow the binding of basic helix–loop–helix proteins with potential repressor activity through Notch signaling [82]. Activation of the Notch receptor cleaves and releases the Notch intracellular domain which migrates to the nucleus to form a transcriptional complex with the DNA-binding protein RBPJ (recombining binding protein suppressor of hairless) [83,84]. The canonical targets of RBPJ include the HES and HEY families of transcriptional repressors, which are basic helix–loop–helix proteins [85,86,87]. Transcriptional repressors of the HES family (HES1–7) bind to N-box promoter regions of their target genes, while repressors from the HEY family (HEY1, HEY2 and HEYL) are associated with E-box promoter regions [86]. In zebrafish, it is known that Fsh stimulates Notch signaling [63]. Therefore, we speculate that Fsh nullified the Gdnf-increased *gdnfa* expression through the Notch pathway and transcription repressors HES and HEY, which would bind to E- and N-boxes within the zebrafish *gdnfa* promoter region. Functional studies of the *gdnfa* promoter region are required to elucidate how Fsh and Gdnf regulate the expression of *gdnfa* in zebrafish testes.

In this study, we demonstrated that *gfrα1a* transcripts were up-regulated by Fsh but not with the same intensity as observed in the Gdnf treatment (a three-fold increase as compared to Fsh). In immature rainbow trout, Bellaiche et al. [31] reported that *gfra1a* mRNA levels were increased following in vitro treatment with Fsh (100 ng/mL—the same concentration as was used in our work). Moreover, the same authors reported that testicular *gfra1a* levels increased towards the end of the reproductive cycle, which coincides with the natural elevation of plasma Fsh levels in rainbow trout [31]. Therefore, in contrast to mammalian species, in which Fsh up-regulated *GDNF*, we have evidence from two teleost species that Fsh modulates the Gdnf-Gfr*α*1 pathway through stimulating, not ligand, but receptor (*gfra1a*) mRNA levels. However, there are some questions that remain. The first concerns whether the Fsh-induced expression of *gfra1a* is mediated by Sertoli cells, germ cells or both. In this work, we have demonstrated that *gfra1a* is expressed by Sertoli and germ cells, while the Fsh receptor is exclusively expressed by somatic cells (Sertoli and Leydig cells) [55]. Therefore, if Fsh-induced *gfra1a* expression is mediated by germ cells, this indicates that the regulation occurs indirectly through growth factors or androgens released by somatic cells (Sertoli and Leydig cells). Moreover, we cannot exclude that the increase in *gfra1a* could also be a consequence of the proliferation of spermatogonia or/and Sertoli cells stimulated by Fsh. More studies are necessary to address the nature of Fsh regulation on *gfra1a* expression in zebrafish testes. Although Gdnf or Fsh independently stimulated *gfra1a* mRNA levels in zebrafish testes, we observed that co-treatment affected negatively the Gdnf-induced expression of *gfra1a*. This is also noted for other genes such as *pou5f3* or *dazl*, whose expressions were higher in the Gdnf treatment as compared to co-treatment with Fsh. For *pou5f3*, a stem cell marker, this observation suggested that Gdnf could be more involved in the maintenance of *stemness* than in increasing the number of stem cells in zebrafish testes. On the contrary, Fsh would be more associated with proliferation towards differentiation, as *pou5f3* was significantly decreased following Fsh co-treatment. Therefore, our data indicate that the pro-differentiating effects of Fsh seemed to be more potent over the stem cell maintenance properties of Gdnf. On the other hand, at the level of differentiation, Gdnf decreased the Fsh effects on spermatogonial differentiation, as the expression of *dazl*, a marker of spermatogonial differentiation, was significantly down-regulated. Altogether, these observations suggest that Gdnf could promote stem cell maintenance through blocking spermatogonial differentiation. This conclusion is also supported by histomorphometrical data showing that Gdnf decreased the frequency of type B spermatogonia and accords with the higher expression of Gfr*α*1a in type A_diff_ spermatogonia.

As Gdnf is a member of the TGF-β superfamily, its role in inhibiting spermatogonial differentiation is likely consistent with other TGF-β superfamily members, such as Amh. Amh is a Sertoli cell growth factor which has been characterized as an inhibitor of spermatogonial differentiation in zebrafish [48,64,87] (see the review in Adolfi et al. [88]). In this regard, we also examined whether Gdnf’s role could be modulated through Amh or by inhibiting Igf3, a pro-differentiation growth factor produced by Sertoli cells [48,54,78,79]. Our data showed that rhGDNF did not modulate either *amh* or *igf3* mRNA levels in the zebrafish testicular explants. Therefore, Gdnf’s role in inhibiting spermatogonial differentiation is not mediated by Amh or Igf3 and it could occur by acting directly on germ cells (autocrine) or indirectly through a different growth factor released by somatic cells (paracrine).

**Figure 10:**
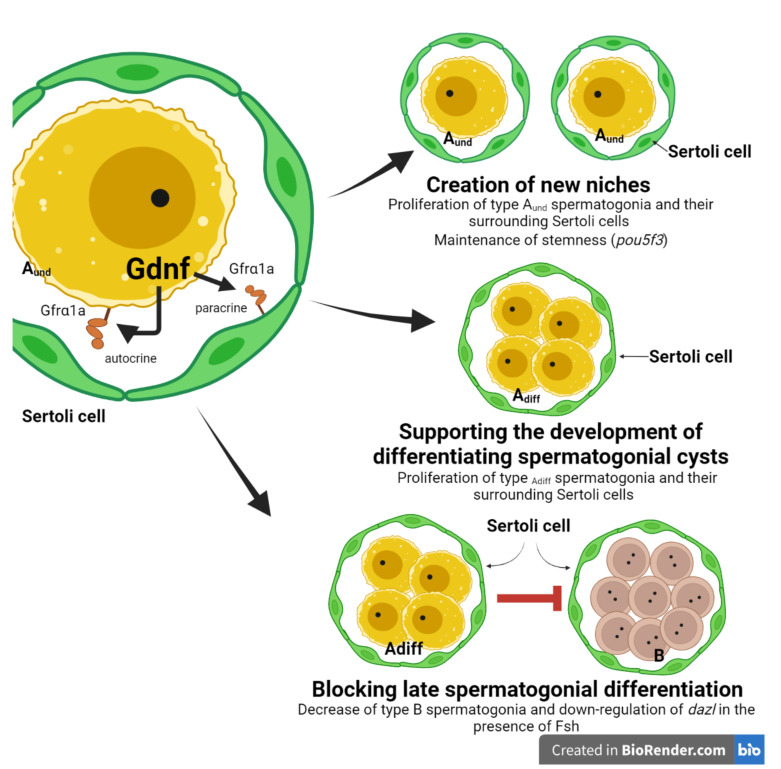
Summary of the effects of Gdnf in the zebrafish spermatogonial niche. Gdnf is a germ cell growth factor which acts on type A spermatogonia and their surrounding Sertoli cells in autocrine- and paracrine-dependent manners, respectively. The Gdnf receptor, named Gfrα1a, is expressed in type A spermatogonia (early spermatogonia, with higher expression in types A_und_ and A_diff_) and Sertoli cells. The main actions of Gdnf are: (1) the creation of new available niches; (2) support of the development of early differentiating spermatogonial cysts; and (3) blocking of late spermatogonial differentiation.

In summary, Figure 10 depicts our main findings regarding Gdnf actions in zebrafish testis. Gdnf is a germ cell growth factor that acts on type A spermatogonia and Sertoli cells in autocrine- and paracrine-dependent manners, respectively. The Gdnf receptor, named Gfr*α*1a, is expressed in type A spermatogonia (highly expressed in types A_und_ and A_diff_) and Sertoli cells. The main actions of Gdnf are: (1) the creation of new available niches by stimulating proliferation of both type A_und_ spermatogonia and their surrounding Sertoli cells. In this context, we highlight that Gdnf stimulates the proliferation of Sertoli cells, which are associated with type A_und_ undergoing mitosis. As a consequence, Gdnf increases the number of available niches and maintains the *stemness* pool in the zebrafish testes; (2) support of the development of differentiating spermatogonial cysts through proliferation of type A_diff_ and their surrounding Sertoli cells; and finally, (3) inhibition of late spermatogonial differentiation, as shown by the decrease in type B spermatogonia and down-regulation of *dazl* in the co-treatment with Fsh. Altogether, our data indicate that the autocrine and paracrine roles of Gdnf are evolutionary novelties in fish, although some paracrine functions are conserved, being similar to those observed for mammalian GDNF.

## Figures and Tables

**Table 1:** Primers used for gene expression analysis (RT-qPCR) and to generate DNA templates for digoxigenin (DIG)-labeled cRNA probe synthesis for in situ hybridization (ISH) (Supplementary Materials).

Target Genes	Primer Sequences (5′–3′)	References
*ef1α*	GCCGTCCCACCGACAAG (Fw)	Morais et al. [48]
	CCACACGACCCACAGGTACAG (Rv)	
*b-actin*	AGACATCAGGGAGTGATGGT (Fw)	Tovo-Neto et al. [49]
	CAATACCGTGCTCAATGGGG (Rv)	
*gdnfa*	GAAGCTCCGGTCTGTATGGA (Fw)	This paper
	GGAGCTCAGGAGCAACAAAC (Rv)	
*gdnfb*	AGGAGTAAATCAGTGGGCCAAA (Fw)	This paper
	AGTAGCTGAATATGAGCTCCTCC (Rv)	
*gfr*α*1a*	TCGACTGGCTCCCATCTATTC (Fw)	This paper
	AGGTGTCATTCAGGTTGCAGG (Rv)	
*gfrα1b*	CCTGTGCTTGATTTAGTGCA (Fw)	This paper
	GCATCCGTACTTTCCCAAAC (Rv)	
*igf3*	TGTGCGGAGACAGAGGCTTT (Fw)	Morais et al. [48]
	CGCCGCACTTTCTTGGATT (Rv)	
*amh*	CTCTGACCTTGATGAGCCTCATTT (Fw)	García-Lopez et al. [50]
	GGATGTCCCTTAAGAACTTTTGCA (Rv)	
*fshr*	GAGGATTCCCAGTAATGCTTTCCT (Fw)	García-Lopez et al. [50]
	TCTATCTCACGAATCCCGTTCTTC (Rv)	
*pou5f3*	GAGAGATGTAGTGCGTGTAT (Fw)	Tovo-Neto et al. [49]
	GCTCGTAATACTGTGCTTCA (Rv)	
*dazl*	AGTGCAGACTTTGCTAACCCTTATGTA (Fw)	Morais et al. [49]
	GTCCACTGCTCCAAGTTGCTCT (Rv)	
*sycp3l*	AGAAGCTGACCCAAGATCATTCC (Fw)	García-Lopez et al. [50]
	AGCTTCAGTTGCTGGCGAAA (Rv)	
*gdnfa-ish*	T7Rpps-CCGCAGTGAGAGCCCCG (Fw)	This paper
	T3Rpps-TCCCGTTAGGTCATATTGTTCCTC (Rv)	

Fw, forward; Rv, reverse; T7Rpps–T7 RNA polymerase promoter sequence at its 5′-end (5′ CCGGGGGGTGTAATACGACTCACTATAGGG-3′), T3Rpps–T3 RNA polymerase promoter sequence at its 5′-end (T3′GGGCGGGTGTTATTAACCCTCACTAAAGGG-3′).

## Data Availability

Data is contained within the article or supplementary material. The data presented in this study are available in this manuscript and supplemental material.

## Supplementary Materials

The following are available online at https://www.mdpi.com/article/10.3390/cells11081295/s1, Figure S1: Morphological characteristics of zebrafish germ cells and *gdnfa in situ* hybridization, Figure S2: Immunofluorescence control using either preadsorbed antibody or omitting the primary antibody, Figure S3: Predicted protein complex models between *Danio rerio* Gfrα1 and rhGNDF (hetero-2-2-mer)., Table S1: Parameters set to reconstruct the phylogeny tree., Table S2: Predicted regulatory binding sites of the GDNF promoter in *Homo sapiens*, *Mus musculus* and *Danio rerio*,Video S1: Interaction between rhGDNF and zebrafish Gfrα1a..
