# Supplementary material for: Gdnf Acts as a Germ Cell-Derived Growth Factor and Regulates the Zebrafish Germ Stem Cell Niche in Autocrine- and Paracrine-Dependent Manners
Source: Cells. 2022 Apr 11;11(8):1295. doi: 10.3390/cells11081295 (PMC9030868; doi:10.3390/cells11081295)
Supplement: Supplementary file 1 [file cells-11-01295-s001.zip › cells-1548641-supplementary (3).pdf]

Supplemental material

1. Material and Methods

1.1 gdnfa expression in zebrafish adult testis

For *in situ* hybridization, a zebrafish *gdnfa*-specific PCR product was generated with primers *gdnfa-ish*-Fw and *gdnfa-ish*-Rv (Table 1). The ~160 bp PCR product was gel purified, and served as a template for digoxigenin (DIG)-labelled cRNA probe synthesis using the RNA labeling (Roche) kit. Gonads were fixed in 4% paraformaldehyde (PFA) in PBS at 4ºC for 2 hours. The protocol used for whole mount (WISH) and in situ hybridization (paraffin embedded) were performed with adaptations, as described previously [1]. Detection of hybridization signal was done with HNPP Fluorescent Detection Set (Roche). Nuclei counter-staining was performed with DAPI (Sigma) (1:10000) diluted in PBS (Phosphate Buffered Saline pH 7.4, sterile-filtered).

2. Results

2.1 Morphological characteristics and in situ hybridization for gdnfa in adult zebrafish testis

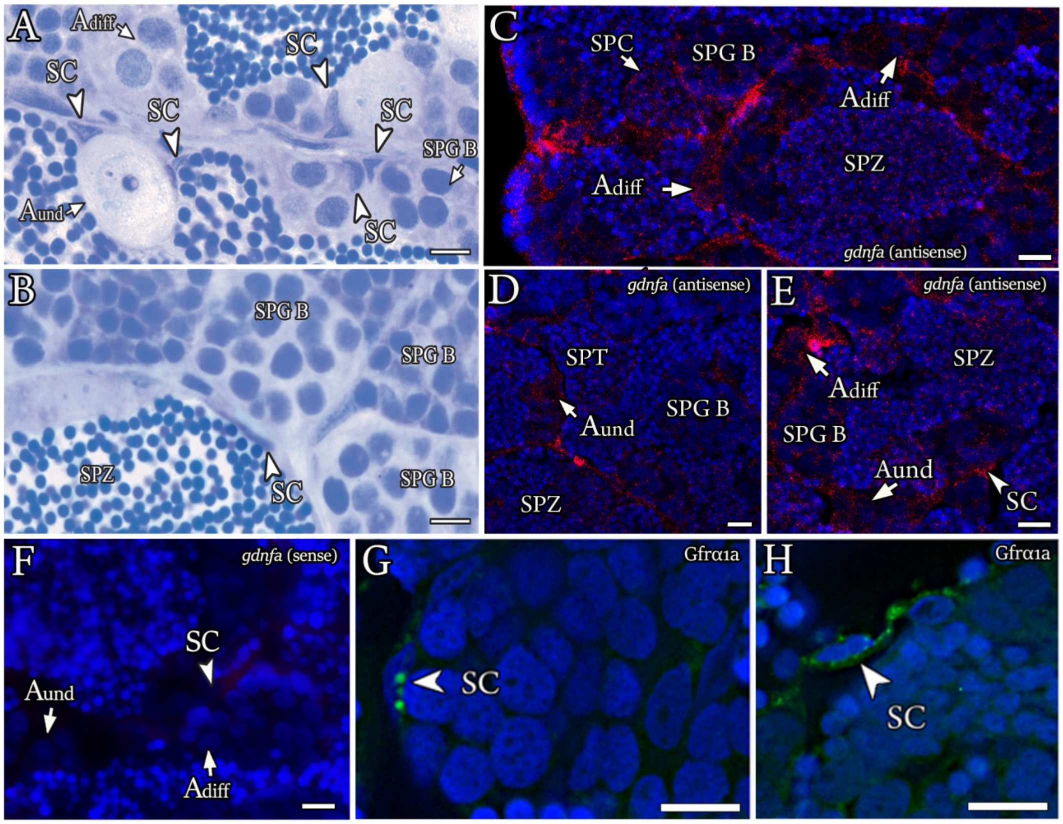

Supplemental Figure S1. Morphological characteristics and *in situ* hybridization for *gdnfa* in adult zebrafish testis. A-B. Adult zebrafish testis section showing different germ cysts (arrow) and Sertoli cells (arrowhead). Scale bars: 5µm. C-E. Detection of *gdnfa* mRNA by *in situ* hybridization. Scale bars: 10 µm. F. *in situ* hybridization using sense (T3) RNA probe. No staining or very weak staining reveals specificity of RNA-antisense probe. Scale bar: 10 µm. G-H. Immunofluorescence for Gfrα1a protein (green) in zebrafish testis. Higher magnification showing Gfrα1a-immunostaining in Sertoli cells (SC). Scale bars: 10 µm. Aund – type A undifferentiated spermatogonia, Adiff type A differentiated spermatogonia, SPG B – type B spermatogonia, SPC – spermatocytes, SPT – spermatids, SPZ – spermatozoa and SC - Sertoli cell.

2.2 Control sections using either preadsorbed antibody with the corresponding peptide or omitting the antibody

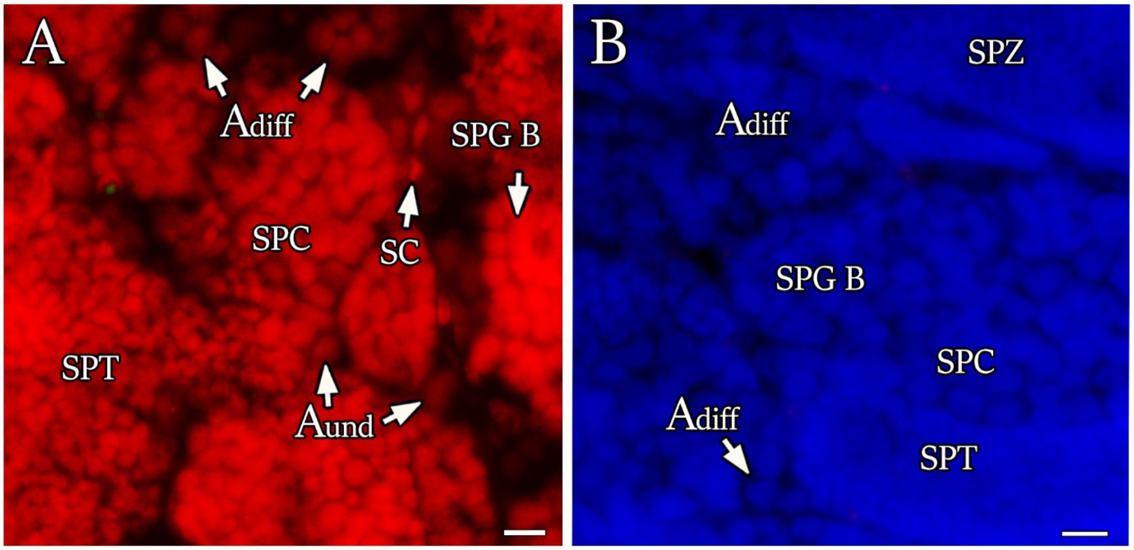

Supplemental Figure S2. Control sections of immunofluorescence of cellular localization of Gfrα1a protein in zebrafish testis (green - A; red – B) using either preadsorbed antibody with the corresponding peptide or omitting the antibody confirming the antibody specificity. Bars - 10 µM. Aund – type A undifferentiated spermatogonia, Adiff type A differentiated spermatogonia, SPG B – type B spermatogonia, SPC – spermatocytes, SPT – spermatids, SPZ – spermatozoa and SC - Sertoli cell.

2.3 Detailed interaction between rhGDNF and the zebrafish receptor Gfrα1a

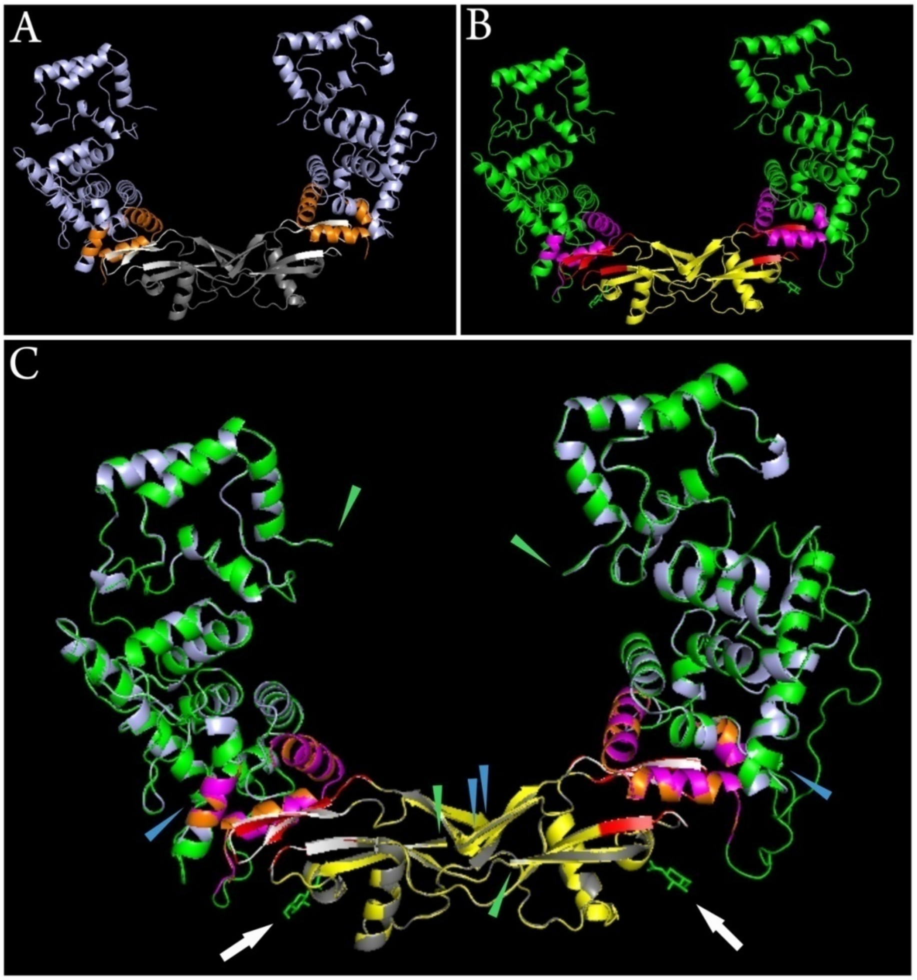

Supplemental Figure S3. Predicted protein complex models of *Danio rerio* Gfrα1 and rhGNDF (hetero-2-2-mer). In A, the template is chosen according to results performed by SWISS-MODEL (swissmodel.expasy.org). Blue arrowheads show the Gfrα1 protein structure and in orange the ligand sites bind to rhGNDF protein. Dark grey represents the rhGNDF proteins and light grey the ligand sites bind to Gfrα1. In B, the 3D model of the Gfrα1-Gndf protein structure. In green, the Gfrα1 protein structure and in purple the ligand sites bind to Gndf protein. Yellow represents the rhGNDF proteins and in red the ligand sites that bind to Gfra1a. Arrows indicating two ligands of N-Acetyl-D-glucosamine. In C, we merged the template and the model of the hetero-2-2-mer showing the similarity of the structures and the identity of the structure formed at the binding sites between Gfrα1 and rhGNDF proteins (orange-purple and light grey-red). Arrows indicating two ligands of the ligand N-Acetyl-D-Glucosamine.

Video 1 here.

Video 1. Video of the detailed interaction between the rhGDNF and its possible ortholog receptor in zebrafish adult testis, Gfrα1a. Despite the evolutionary distance between both receptor and ligand, the ligand is still able to respond to the recombinante human GDNF.

**Bayesiana (Beast v1.7.0)**	
Substitution model	JTT
Base frequencies	estimated
Starting tree	Randomly generated
Generetations/Burn-in	10 000 000/10 000
Sample frequency	1000
Branch support	Posterior probability

Table S1. Parameters set to reconstruct the phylogeny.

**Reference (*Homo sapiens*)**	**Predicted sequence**	**Position related +1**
TATA-box	TATAAA	-33
CRE1	CCTCTGACTTCAGCC	-161
CRE2	GTTTAGGTCAGA	-93
CRE3	GGGCACGTCACGCA	-57
E-box	CATCTG	-388
E-box	CAAGTG	-1294
E-box	CAGTTG	-1694 and -1709
E-box	CACGTG	-1277; -1461 and -1853
NF-kB	GGAGATTCC	-1445
**Reference (*Mus musculus*)**	**Predicted sequence**	**Position related +1**
TATA-box	TATAAA	-33
CRE1	CCTCTGACTTCAGCC	-161
CRE2	GTTTAGGTCAGA	-93
CRE3	GGGCACGTCACGCA	-57
E-box	CAACTG	-805 and -1394
E-box	CAGATG	-1587 and -1865
E-box	CAGTTG	-1690

N-box	CACCTG	-1373; -1451 and -1843
N-box	CACCAG	-1660
AR	TCTTCCAGAACACATACTCCCCAACAA	-829
NF-kB	GGAGATTCCG	-1435
NF-kB	AGTGGCCTT	-1480
**Reference (*Danio rerio*)**	**Predicted sequence**	**Position related +1**
TATA-box	TTAAAAAGCGC	-28
GC-box	GGGCGG	-75
CRE canonical	TGACGTCA	-59
E-box	CAGTTG	-350
E-box	CAACTG	-705; -1411 and -1777
N-box	CACCAG	-1696
AR – half site	AGAACA	-1027 and -1805

Table S2. Predicted regulatory binding sites of the GDNF promoter in *Homo sapiens*, *Mus musculus* and *Danio rerio*.

3. Reference

1. Thisse, C.; Thisse, B. High-resolution in situ hybridization to whole-mount zebrafish embryos. *Nat Protoc*. **2008**, 3, 59-69.
